# Methotrexate plus low-dose prednisolone compared with high-dose corticosteroid therapy in the management of idiopathic granulomatous mastitis

**DOI:** 10.1007/s00404-025-08220-2

**Published:** 2025-11-18

**Authors:** Leyla Shojaee, Nahid Nafissi, Fatemeh Niksolat Roodposhti, Kiana Shakeriastani, Kiarash Shakeriastani

**Affiliations:** 1 Department of Surgery, School of Medicine, Mazandaran University of Medical Sciences, Sari, Iran; 2 Breast Health & Cancer Research Center, Iran University of Medical Sciences, Tehran, Iran; 3 Orthopedic Research Center, School of Medicine, Mazandaran University of Medical Sciences, Sari, Iran; 4 Concordia University, Montreal, Canada; 5 Wake Forest Institute of Regenerative Medicine, Winston-Salem, NC USA

**Keywords:** Idiopathic granulomatosis mastitis, Recurrent, Treatments

## Abstract

**Purpose:**

Idiopathic granulomatous mastitis (IGM) is a chronic, relapsing inflammatory breast disease of unknown etiology. Its unpredictable course and unclear pathogenesis have made treatment a persistent clinical challenge. This study aimed to evaluate treatment outcomes in patients with IGM, focusing on extending remission periods and reducing relapse rates after discontinuation of therapy.

**Methods:**

This retrospective cohort study included 200 patients with histopathological confirmed idiopathic granulomatous mastitis who were referred to the Teaching and Therapeutic Center at the Central Hospital of Mazandaran University of Medical Sciences. Patient demographics, pathological characteristics, treatment regimens, remission duration, and recurrence rates were analyzed.

**Results:**

From January 2018 to December 2024, a total of 200 patients were enrolled in the study, with a mean age of 33 years (range: 18–54 years) and a mean follow-up duration of 34 months (range: 8–51 months). Patients were divided into two treatment groups: Group A received high-dose prednisolone combined with abscess drainage (*n* = 99), while Group B received low-dose prednisolone, abscess drainage, and methotrexate (*n* = 101). Group B demonstrated a significantly shorter treatment duration (11.56 ± 2.15 months) compared to Group A (14.86 ± 3.42 months; *P* < 0.001). The mean time to follow-up was significantly longer in Group B (35.97 ± 6.96 months) than in Group A (20.00 ± 7.02 months; *P* < 0.001). The recurrence rate was lower in Group B (4.9%) than in Group A (23.2%), with the difference approaching statistical significance (*P* = 0.057). In addition, the mean time to relapse in Group B (34.46 ± 6.50 months) was significantly longer than that of Group A (16.96 ± 5.34 months), indicating that patients receiving the combined regimen of low-dose prednisolone, drainage, and methotrexate experienced a more sustained remission period.

**Conclusions:**

According to the results, the treatment of idiopathic granulomatous mastitis, especially the moderate to severe form of the disease, using combination therapy with low-dose prednisolone and methotrexate can be associated with a reduction in treatment duration, and increase in remission duration, and a reduction in relapse, and drug side effects.

## What does this study add to the clinical work

**Table:** 

Early initiation of low-dose methotrexate with corticosteroids significantly reduces treatment duration in patients with idiopathic granulomatous mastitis (IGM). Methotrexate-based therapy prolongs relapse-free remission and lowers recurrence rates compared to high-dose corticosteroids. Patients treated with methotrexate experience fewer drug-related complications, enhancing treatment tolerability and patient outcomes.	

## Introduction

Granulomatous mastitis is a chronic, benign inflammatory disease of the breast, thought to have an autoimmune etiology. Due to its unclear pathogenesis, multiple therapeutic approaches have been employed [1] Nonetheless, frequent recurrence and inadequate response to existing therapies continue to pose significant challenges for affected patients [2].

Idiopathic granulomatous mastitis (IGM) predominantly affects women of childbearing age [2]. Factors such as oral contraceptive pill (OCP) usage, elevated prolactin levels, and breast-feeding are implicated in initiating and perpetuating inflammation within breast ducts [3]. Clinical manifestations of IGM vary considerably among patients [4]. In some cases, the disease remains mild, confined primarily to a localized region around milk ducts. In others, it presents extensively, involving multiple areas within the breast [5].

Treatment response also varies significantly among individuals. Some patients achieve resolution with antibiotics or conservative interventions, such as drainage of abscesses [6]. Others, however, require second-line therapies involving immunosuppressive agents, including low- or high-dose corticosteroids, and occasionally surgical intervention [7]. Patients who exhibit resistance to these therapies or develop complications may necessitate third-line immunosuppressive agents, such as azathioprine or methotrexate [8]. Despite clinical improvements, relapses frequently occur after treatment cessation, significantly affecting patient outcomes [9].

Given the variability in clinical presentations and treatment responses, further investigation into the efficacy and outcomes of current therapeutic strategies is essential. Therefore, this study aimed to evaluate treatment outcomes in patients diagnosed with IGM presenting with moderate to severe disease, with the goal of identifying effective approaches and reducing the incidence of recurrence.

## Methods

This retrospective observational cohort study was conducted at the Teaching and Therapeutic Center of Mazandaran University of Medical Sciences between January 2018 and December 2024. A total of 200 female patients with histopathological confirmed idiopathic granulomatous mastitis were enrolled following their initial presentation with clinical features, such as breast pain, erythema, edema, or palpable mass. Inclusion criteria comprised women aged 18 years or older with no prior history of malignancy, trauma, or infectious mastitis, and who provided informed consent for participation. Diagnosis of IGM was established through a combination of clinical examination, breast ultrasound, and core needle biopsy interpreted by experienced pathologists.

Patients were evaluated and managed by their treating physicians based on routine clinical judgment and institutional protocols. Treatment decisions, including drug regimens, were not influenced or assigned by the investigators but were observed as part of standard clinical care. The study team systematically recorded demographic data, clinical findings, disease extent, relevant medical history, and therapeutic approaches employed by clinicians.

### Data collection and variables

Comprehensive baseline data were collected at the time of enrollment, including patient demographics (age, BMI), obstetric history (pregnancy and breast-feeding), history of autoimmune or rheumatologic disease, oral contraceptive use, smoking status, and extent of breast involvement (unilateral vs. bilateral, number of quadrants involved). Disease severity and clinical characteristics at presentation were assessed and recorded using standardized forms by trained clinical staff.

Patients were grouped retrospectively based on the treatment regimen chosen by their managing physicians. Group A included patients who received high-dose corticosteroids, while Group B comprised those treated with low-dose corticosteroids and methotrexate as first line treatment.

Patients in Group A received treatment with oral corticosteroids (prednisolone) at an initial daily dose of 1 mg/kg and effective drainage of breast abscesses was performed as clinically indicated.

Patients in Group B received low-dose prednisolone at a daily dose of 10 mg, combined with methotrexate at an initial dose of 10 mg weekly along with folic acid supplementation. The methotrexate dosage was increased to 15 mg weekly based on clinical response. Abscess drainage procedures were similarly performed when clinically necessary.

For patients in Group A, the dose of prednisolone was gradually tapered every 4–6 weeks following demonstration of adequate clinical improvement. In Group B, following a satisfactory response to therapy, the methotrexate dose was reduced by 5 mg every 3 months, while prednisolone was tapered by 2.5 mg every 3 months.

### Follow-up and outcome measures

Participants in both groups were regularly evaluated every 4–6 weeks by a multidisciplinary team consisting of rheumatologists and surgeons. Clinical assessments at each visit included evaluation of disease severity, response to treatment (assessed by reduction in pain and breast lesion size), and careful monitoring for medication-related adverse events, such as hypertension, weight gain, bone pain, and gastrointestinal disturbances.

Laboratory monitoring, including complete blood count, blood glucose levels and liver enzyme tests, was conducted every 3 months. Concurrently, ultrasound examinations of the breast were performed to objectively assess treatment efficacy and changes in disease extent.

The primary outcome measures were remission and relapse rates observed during the follow-up period. Data regarding treatment effectiveness, medication side effects, and patient outcomes were systematically documented and analyzed.

### Statistical analysis

Data were analyzed using SPSS V.24. For the analysis of quantitative values in two groups, *t* test and chi-square test were used for analyzing two qualitative variables, and if the sample size in the group was small, Fisher's exact test was used. For time to relapse, Kaplan–Meier curves and log-rank test were used. Relapse rates were also standardized according to follow-up times. To examine the relationship of study variables with recurrence, the logistic regression model was applied.

## Results

### Patient demographics and study duration

A total of 200 patients diagnosed with granulomatous mastitis were enrolled in this study and underwent diagnostic and therapeutic evaluation, as well as follow-up, from January 2018 to December 2024. The mean age of patients was 33 years (range: 18–54 years), and the average follow-up duration was 34 months (range: 12–54 months). There was no statistically significant difference in age between the two groups; both had a mean age of 33 years (Table 1).

**Table 1:** Baseline characteristics of patients included into the study

Variable	Group A (*N* = 99)	Group B (*N* = 101)	*P* value
Age (years)	34.03 (21–55)	33.16 (22–52)	0.379
BMI			
< 30	54.5%	37.6%	0.481
≥ 30	45.4%	62.4%	
Lesion location			
Unilateral breast	73.7%	84.2%	0.700
Bilateral breast	26.3%	15.8%	
≤ One quadrant	32.7%	27.3%	0.556
> One quadrant	76.3%	72.7%	
Clinical presentation			
Painful mass	46.5%	63.4%	< 0.001
Erythema and edema	73.0%	34.7%	< 0.001
Fistula	76.7%	78.5%	0.605
History of autoimmune disease			
Negative	87.9%	86.1%	0.834
Positive	12.1%	13.9%	
History of pregnancy			
Negative	6.1%	3%	0.721
Positive	93%	98%	
History of breast-feeding			
Negative	6.1%	3%	0.850
Positive	93.9%	97%	
Cigarette use			
Negative	7%	8.9%	0.522
Positive	93%	91.1%	
OCP use			
Negative	10.1%	15.8%	0.826
Positive	89.9%	84.2%	

### Clinical presentation

The most common clinical presentation was a painful breast mass, observed in 63.4% of patients in Group B and 46.5% in Group A. Erythema was present in 73.0% of Group A and 34.7% of Group B. Fistula and ulcer formation were reported in 67.7% of Group A and 78.5% of Group B. Most patients had a history of pregnancy and breast-feeding. Only 6 patients in Group A and 3 patients in Group B were single and had no history of either (Table 1). The mean number of full-term pregnancies among patients was 1.52, with a median of 1 in both treatment groups.

### Risk factors and comorbidities

The history of oral contraceptive pill (OCP) use was similar across both groups, with the majority of patients having no history of OCP use. The extent of disease, defined as involvement of one or more quadrants of the breast, was observed in 72.7% of Group A and 76.3% of Group B, showing no significant differences in disease severity was observed between the two groups.

A history of autoimmune or rheumatic disease was reported in 12.1% of Group A and 13.9% of Group B, with no significant intergroup difference. Similarly, there was no difference in smoking history, breast-feeding and BMI between the two groups (Table 1).

### Treatment duration and relapse

The mean treatment duration was shorter in Group B (11.56 ± 2.15 months) compared to Group A (14.86 ± 3.42 months). Following treatment, the mean follow-up period in Group B and A was 35.97 ± 6.96 and 20.00 ± 7.02 months, respectively.

Relapse occurred in 4.9% of patients in Group B, who received low-dose prednisolone combined with methotrexate, compared to 23.2% in Group A, who were treated with high-dose prednisolone alone (*P* = 0.057). Mean time to relapse in group B was significantly higher than group A (34.46 ± 6.50 vs. 16.96 ± 5.34).

To account for differences in follow-up duration, relapse rates were standardized per 100 person-months. The control group (Group A) exhibited a relapse rate of 1.16 per 100 person-months, whereas the treatment group (Group B) had a substantially lower rate of 0.36 per 100 person-months, indicating a significantly reduced standardized relapse rate in the B group (Table 2).

**Table 2:** Mean treatment duration and relapse in study groups

Variable	Group A	Group B	*P* value
Treatment duration (months)	14.86 ± 3.42	11.56 ± 2.15	< 0.001
Time to relapse (months)	16.96 ± 5.34	34.46 ± 6.50	< 0.001
Crude relapse rate (%)	23.2 (14.9–21.5)^†^	4.9 (0.7–9.1)^†^	0.057
Standardized relapse rate	1.16 + 0.24* per 100 person-months	0.36 + 0.10* per 100 person-months	0.002

*Standard Error; ^†^Confidence interval 95%

**Fig. 1:**
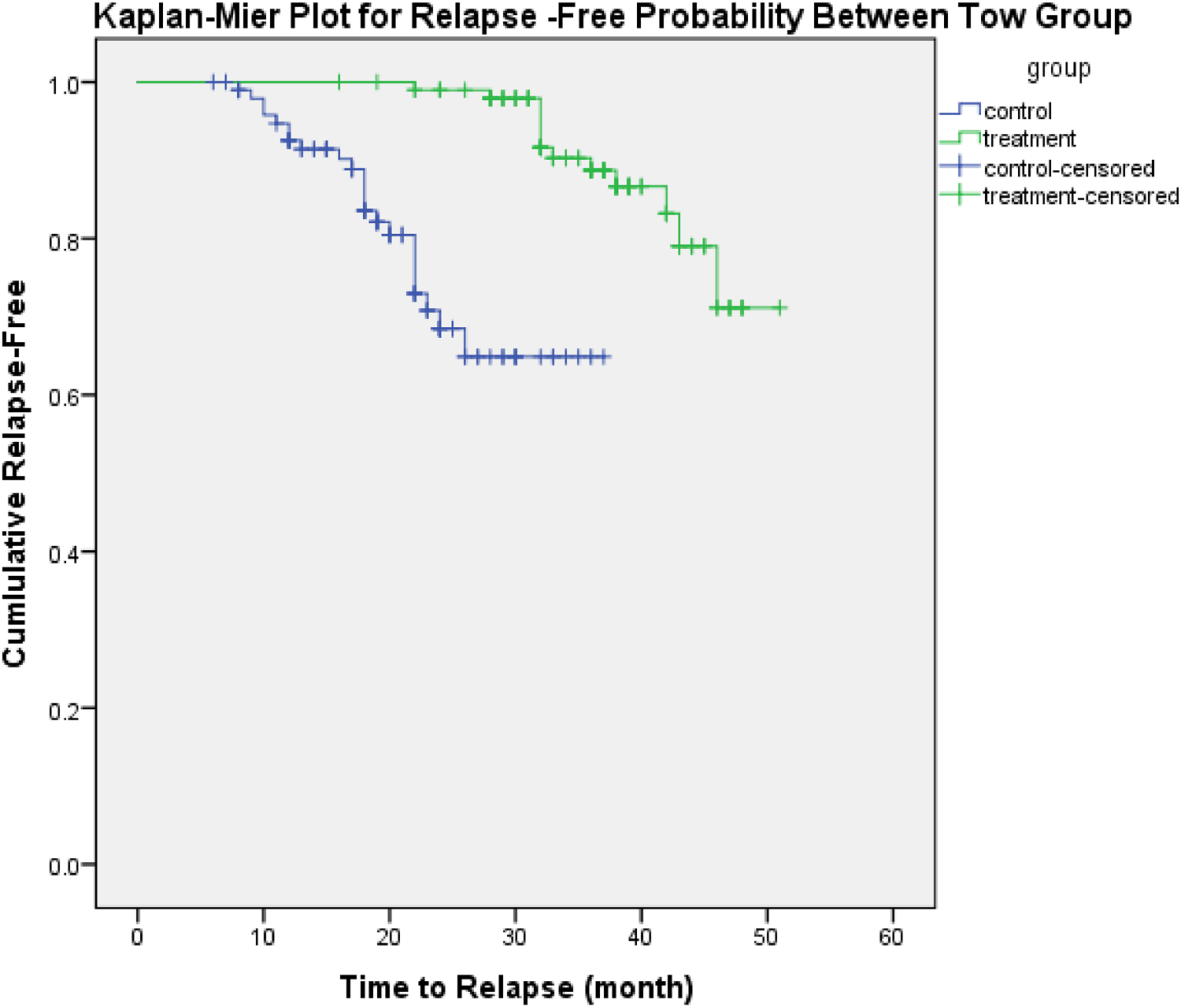
Kaplan–Meier curve on time to relapse between study group

The Kaplan–Meier survival curve clearly demonstrates a significant difference in time to relapse between the two treatment groups. Patients receiving methotrexate in combination with low-dose prednisolone exhibited a longer relapse-free survival compared to those treated with high-dose alone (Fig. 1).

### Logistic regression analysis

In the logistic regression analysis, no significant association was observed between recurrence of disease and variables, including BMI, OCP use, cigarette smoking, disease extent, side of involvement, or history of autoimmune disease. The treatment group with methotrexate plus low-dose prednisolone was also associated with lower odds of recurrence (OR = 0.488, 95% CI 0.231–1.029); however, the association was not statistically significant in the enter model (Table 3). In the backward logistic regression model, treatment type showed a borderline association with the outcome (*P* value = 0.05). Although not statistically significant at the conventional 0.05 level, both models suggested a trend toward improved outcomes in the treatment group.

**Table 3:** Logistic regression analysis of risk factors associated with recurrence

Variable	Odds Ratio (OR)	95% CI	*P* value
≤ One quadrant involvement	0.835	0.318–2.195	0.715
Unilateral breast involvement	1.253	0.527–2.978	0.610
Positive history of autoimmune disease	0.530	0.145–1.941	0.338
Treatment Group B (MTX + low-dose prednisolone)	0.456	0.177–1.174	0.104
BMI < 30	1.172	0.555–2.476	0.677
Cigarette smoking	0.843	0.175–4.053	0.831
Contraceptive use	1.049	0.354–3.107	0.932

### Drug-related complications

Drug-related complications were nearly twice as frequent in Group A (51.5%) compared to Group B (23.8%), representing a statistically significant difference (*P* value < 0.001). Hyperglycemia (43% in A group, 10% B group), gastritis (56% in A group, 13% B group), weight gain (69% in A group, 7% B group), bone pain (48% in A group, 6% B group), and cushing face (78% in A group, 0 B group). More serious side effects of methotrexate use include myelosuppression, hepatotoxicity, and an increase in creatinine and hair loss have not been observed in patients receiving it. Most of the patients in this study are young and did not experience any side effects that required discontinuation of the drug after taking methotrexate and folic acid. In a few cases, nausea was observed while receiving injectable methotrexate, for which the patient was given an antiemetic drug.

## Discussion

The treatment of IGM remains a significant clinical challenge. Due to its unclear etiology, there is currently no standardized or definitive treatment protocol. Various therapeutic approaches, such as antibiotics, corticosteroids, and surgery, have been proposed [10].

The severity of the disease is classified into grades 1 to 4 based on inflammatory changes in the skin and soft tissues, accumulation of abscesses and skin fistulas, destructive changes in the skin and formation of ulcers, as well as the presence or absence of extramammary symptoms. The disease in grades 1 and 2, which have mild symptoms and no skin destruction and extramammary symptoms, is controlled with symptomatic treatment, and the use of NSAIDs and antibiotics; however, in stage 3, the disease is defined as severe but does not have extramammary symptoms, and stage 4 disease is defined as having extramammary symptoms with any degree of inflammation. In these patients, treatment with corticosteroids and antimetabolites such as methotrexate or azathioprine is used [11].

In another study, the severity of the disease is defined based on the extent of inflammatory involvement of the breast, such that if less than 10% of the breast is involved, the stage is mild, if between 10% and 25% of the breast is involved, the stage is moderate, and if more than 25% of the breast is involved, the severe type of the disease is defined [12]. In this study, patients with mild and moderate involvement received treatments, such as antibiotics, abscess drainage, and immunosuppressive therapy, and in severe cases, surgical intervention was done. Immunosuppressive therapy was used in 74% (83 patients), including intralesional steroid injections in 43 patients. Patients with severe symptoms underwent surgical interventions more frequently than patients with moderate and mild symptoms (21.4% vs. 0% and 7.5%, respectively; *p* = 0.004).

However, patients frequently experience high relapse rates or suffer adverse effects associated with prolonged antibiotic or corticosteroid therapy. Even among those who initially respond well to long-term treatment, disease recurrence often occurs shortly after cessation of therapy.

In this study, most patients based on the classification of the extent of breast involvement, inflammatory symptoms such as edema and erythema or painful mass were observed in more than one quadrant (> 25%) of the breast. In fact, about over 70% of patients in both groups suffered from the severe type of the disease; therefore, the decision-making regarding the type of treatment to control symptoms earlier and to provide better treatment with fewer side effects and a lower likelihood of recurrence was considered.

Several studies have demonstrated the potential benefits of methotrexate in reducing disease relapse, mitigating inflammation, and minimizing treatment-related side effects. These improvements are largely attributed to the corticosteroid-sparing effects of methotrexate, allowing for reduced steroid dosage [11–13]. In one study, methotrexate was administered to 28 patients and resulted in the highest remission rate with a relapse rate of only 13.2% [14]. Another study reported that 80% of patients treated with methotrexate, 42% of those receiving corticosteroids alone, and 66% of those treated with corticosteroids plus surgery experienced sustained remission [13].

A systematic review by Sarmadian et al. further highlighted the variable relapse rates associated with different treatment modalities: 65% in patients treated with drainage alone (across four studies), 11% among those receiving steroids and antibiotics (six studies), 23% among those undergoing surgery with steroid resection (six studies), and 13% following methotrexate use (five studies). The lowest relapse rate was observed in patients receiving a combination of methotrexate and corticosteroids [15].

Traditionally, prednisolone is considered a second-line treatment, while methotrexate and azathioprine are introduced as third-line options—typically in cases, where initial therapies (e.g., antibiotics, drainage, or corticosteroids) fail or cause intolerable side effects or relapse [16–19]. In this study, patients who were unwilling to start high-dose corticosteroids due to side effects, were prescribed with low-dose prednisolone in addition to methotrexate. This strategy facilitated more rapid clinical improvement, especially among patients in Group B, who tolerated the low-dose prednisolone and methotrexate regimen well and experienced fewer side effects.

Our findings suggest that the addition of methotrexate significantly shortens treatment duration and prolongs remission. Furthermore, in the few patients who did experience relapse, the interval between remission and recurrence was longer in the methotrexate group, indicating more sustained disease control. A study by Kazem Senol et al. supports these findings, reporting lower relapse rates in patients treated with low-dose corticosteroids and methotrexate compared to those receiving surgery, corticosteroids alone, or corticosteroids combined with azathioprine [20]. Similarly, Schmajuk et al. demonstrated that methotrexate monotherapy, administered weekly at moderate doses, effectively controlled the disease, though they recommended further investigation into optimal dosing and treatment duration [21].

In this study, the crude relapse rate was 23.2% in group A and 4.9% in the group B. Notably, the incidence of treatment-related side effects was significantly higher in the group A (51.5%) compared with group B (23.8%). Most of these complications were linked to the use of high-dose corticosteroids. The side effects of methotrexate consumption are significantly lower than those of high-dose prednisone, and patients had better tolerance during continued treatment.

Although the crude relapse rates appeared different between the two treatment groups, the difference did not reach statistical significance (borderline significance, *P* = 0.057). This finding can be explained by the relatively small number of relapse events in our cohort, the fact that several patients remained censored at the end of follow-up, and the limited study period, which prevented the Kaplan–Meier curve from reaching the median survival point. These factors likely reduced the statistical power to detect a significant difference. Nevertheless, when relapse was standardized by duration of follow-up, patients receiving methotrexate plus low-dose prednisolone demonstrated a markedly lower relapse rate compared with those treated with high-dose corticosteroids. This trend, although not statistically significant, suggests a potential clinical benefit of the combined regimen and highlights the need for larger prospective studies with longer follow-up to confirm these findings.

Following the administration of prednisolone, especially at high doses, patients may experience several side effects. The most common and important side effects include digestive problems (dyspepsia, stomach ulcers), weight gain, hair loss, osteoporosis and bone pain, and increased blood sugar [22]. For this reason, all patients are given dietary recommendations in the form of calorie control by reducing carbohydrate intake and reducing the consumption of high-fat foods, and performing sports activities. If patients have serious problems with high blood sugar, digestive problems, and drug intolerance, the drug dose is adjusted, and blood sugar control is achieved with the help of medication and diet, and digestive problems are controlled by adjusting the dose of prednisolone and taking antacids [23]. In addition, patients taking methotrexate may experience side effects, such as bone marrow suppression.Increased liver enzymes, hair loss, and less common side effects such as drowsiness, pulmonary complications, mouth ulcers, and blurred vision may occur. For this reason, patients regularly undergo tests including CBC, LFT, BUN, and CR every 3 months. If liver enzymes increase more than twice, or WBC, HG, and PLT decrease, the drug is withheld and the patient is monitored. If there is improvement, treatment is resumed with an adjusted dose [24].

This study underscores the potential value of initiating methotrexate-based therapy earlier in the treatment course for patients with extensive disease. Nevertheless, larger studies and multicenter trials are needed to validate these findings, optimize treatment strategies, and further reduce relapse rates in patients with idiopathic granulomatous mastitis.

## Conclusion

Methotrexate plus low-dose corticosteroids was associated with a lower crude relapse rate and fewer steroid-related adverse effects compared with high-dose corticosteroids in patients with idiopathic granulomatous mastitis.

## Data Availability

No data sets were generated or analyzed during the current study.
